# Reasons women terminate their pregnancies legally and their contraceptive practices at Soshanguve 3 Community Health Centre, Tshwane district, South Africa

**DOI:** 10.4102/safp.v62i1.4310

**Published:** 2020-03-26

**Authors:** David K.K. Masanabo, Indiran Govender, Tombo Bongongo

**Affiliations:** 1 Department of Family Medicine and Primary Health Care, Faculty of Health Sciences, Sefako Makgatho Health Sciences University, Pretoria, South Africa; 2 Department of Family Medicine, University of Pretoria and Kalafong Hospital, Pretoria, South Africa

**Keywords:** reasons for legal TOP and contraceptive practices, Soshanguve, South Africa, unplanned pregnancy, limiting childbearing, socio-economic problems

## Abstract

**Background:**

Various reasons have been cited in studies conducted in South Africa on why women legally terminate their pregnancies. We sought to determine the reasons for women to terminate their pregnancies legally and their contraceptive practices. This study was conducted at Soshanguve 3 Community Health Centre (CHC), located in a semi-rural zone in the north-west of Pretoria, Gauteng province of South Africa.

**Method:**

A cross-sectional study design was adopted in this study.

**Results:**

Of the 250 respondents, high participation (23.2%) was noted amongst women aged 18–20 years. Eighty-three (33.2%) respondents did not have children, 108 (43.2%) had completed their secondary school education and 226 (90.4%) were Christian. Of the participants, 80% were single and 62.8% were unemployed. About 85.6% (214) of respondents had not had a previous abortion. A total of 24% of respondents requested abortion because they wanted to focus on their education, while 23.1% were not ready to be parents and 21.7% experienced financial difficulties. With regard to practice, all respondents had already used contraception and the most used contraceptive was the male condom (43.5%), followed by an injectable contraceptive (7.1%).

**Conclusion:**

While academic reasons, not being ready to be a parent and financial difficulties were named as the main reasons for terminating a pregnancy legally, the selected pregnant women at Soshanguve 3 CHC demonstrated an unsatisfactory practice of contraceptive measures.

## Introduction

Induced termination of pregnancy (TOP) is defined as the separation and expulsion of the contents of the uterus of a pregnant woman by medical or surgical means.^1^ It is one of the most commonly performed gynaecological procedures in the world, with about 41.6 million induced TOPs performed in 2003 and about 46 million in 2013.^2,3^ Women seeking legal TOP usually report that the pregnancy was unplanned or unwanted.^4^ They provide various reasons for seeking legal TOP, such as financial difficulties, not being in a committed relationship, partner-related problems and having completed their families. These reasons, in turn, are influenced by different circumstances (such as social, economic and health issues) that surround their TOP decision-making.^4^ A review study on 14 countries (both developed and developing countries) revealed that socio-economic concerns or limiting childbearing were the most frequently cited reasons for seeking TOP.^5^ In a United States-based study, 40% of the respondents who presented for legal TOP stated financial instability as their main reason for doing so.^6^ In addition, socio-economic concerns were also reported by 32% of Swedish women and 23% of Belgian women who presented for TOP at designated TOP facilities.^5^ These findings emphasise the widespread influence of socio-economic circumstances in women’s reproductive decision-making. It is thus ranked high in the reasons that women provide for seeking TOP.

In Africa, where access to safe and legal TOP is still a challenge, an estimated 30% of pregnancies end in TOP annually.^6^ In an Ethiopian study on women seeking TOP, 36.7% of the respondents reported socio-economic problems as their reason for TOP, while 8.1% of women reported that they wanted to complete their education before having children and another 8.1% of the respondents reported partner pressure or influence as the main reason for requesting TOP.^7^ Similarly, financial instability as the main reason for TOP was mostly cited (by 20.9% of women) in a Nigeria-based study on women seeking TOP.^8^ In addition, socio-economic factors were ranked high in a Congo, Ghana and Gabon-based study.^5^ A substantial proportion of TOPs in these studies occurred amongst young, unmarried women with no or inadequate financial means to raise a child. As a result, TOP becomes the best option to avoid an unplanned or unwanted pregnancy.

In South Africa, 30% – 50% of women present with an unwanted and unplanned pregnancy, which is ultimately terminated.^9^ Termination of pregnancy is legal under the *Choice on Termination of Pregnancy Act* (Act No. 92 of 1996), which was amended in 2004 (Act No. 38 of 2004) and again in 2008 (Act No. 1 of 2008).^4^ Pregnant women have been utilising this service to terminate unplanned pregnancies and between 2012 and 2013 almost 90 000 TOPs were performed in state clinics and hospitals.^4^ A variety of reasons have been cited in several studies that aimed to determine the reasons for women to terminate their pregnancies legally. Socio-economic problems as the main reason for requesting TOP were reported by 96.1% of respondents in a KwaZulu-Natal-based study.^9^ Other studies found that women terminate pregnancies legally because of inappropriate timing of the pregnancy and partner-related problems.^4,10^ Most of the women terminating their pregnancies in South Africa were found to be young women between the ages of 20 and 30 years.^11^ In Hammanskraal, South Africa, 36.1% of the women who chose TOP were single, widowed and had at least one child; 28% were high school learners, 46.4% had completed secondary school education; and 35.5% had no formal education. The majority of these women (73.5%) seeking TOP were unemployed.^4^ These are women who fall prey to male dominance because of the lack of economic empowerment and financial independence and thus are dependent on men for support.^12^

Contraceptive practice in women seeking TOP has been studied in various relevant literatures, and the outcome indicates that knowledge of contraception does not necessarily result in the correct and regular use of contraceptives.^4^ In south-western Nigeria, 91.7% of women who had one or more TOPs had knowledge about contraceptives, but only 21.5% reported to have used a contraceptive at their first intercourse after they had had TOP.^8^

In South Africa, the knowledge of contraceptive measures does not correspond with the practice or the use of contraception, as demonstrated in a cross-sectional study conducted.^13^ Although the practice of contraception was 44.1%, in the same sample, knowledge about contraception was 85.8%. Looking at contraceptive use, it was found that emergency contraceptives, male condoms, oral contraceptive pills and injectable contraceptives were commonly cited methods.^13^ Ineffective use and non-utilisation of contraceptives result in unplanned and unwanted pregnancies in women of reproductive age who are sexually active. These women resort to TOP.^4^

This study aimed to determine the reasons for women to terminate their pregnancies legally and their contraceptive practices at Soshanguve 3 Community Health Centre (CHC), Gauteng province, South Africa.

## Methodology

### Study design and setting

This was a cross-sectional study using a self-administered standardised questionnaire. The study was conducted at Soshanguve 3 CHC, which is located in a semi-rural zone north-west of Pretoria, in the Gauteng province of South Africa.

### Study population

Only pregnant women from 18 years of age and above were targeted for the study. With respect to age, the estimated number of pregnant women attending Soshanguve 3 CHC for TOP on a monthly basis was 42. The time frame assigned to this study was 6 months. The expected population of pregnant women was around 252. A convenience sampling was applied and 250 respondents (99.2% of our expected population) were recruited during the proposed time frame.

### Data collection

A self-administered standardised questionnaire on TOP was used. This questionnaire was developed and used in Ontario, Canada.^14^ It was also used in Sweden, Russia, Britain and South Africa. The English standardised questionnaire was translated into Setswana by a qualified translator as these are the two languages spoken in the study area. All pregnant women seeking TOP at the Soshanguve 3 CHC were introduced to the study by an assistant who had been trained by the principal author. Only those who consented to take part in the study were recruited and given the questionnaire. The trained assistant helped the respondents on how to complete the questionnaire.

### Data analysis

Raw data were captured in a Microsoft Excel spreadsheet. All statistical analysis was performed using Statistical Analysis Software version 9.4. Associations were tested for significance using Fisher’s exact test. A confidence interval of 95% was used while reporting the results and a *p*-value of 0.05 or less was considered significant. The results of the study were presented in the form of frequencies and percentages summarised in tables from which interpretations were made.

### Ethical considerations

Permission to conduct the study was obtained from the Ethics Committee of Sefako Makgatho Health Sciences University (reference number: SMUREC/M/150/2017: PG) and also from the Operational Manager of Shoshanguve 3 CHC. Written informed consent was obtained from each participant and confidentiality and anonymity were maintained throughout the entire research process. The participants were informed of their rights to withdraw from the study at any stage of the research process if they felt uncomfortable.

## Results

The highest percentage of participation was 23% and was noted in the age category of 18–20 years. A total of 36% of respondents had one child, while 43% had completed secondary school education. Of the respondents, 90% were Christian and 80% were single. A total of 70% of respondents were unemployed and 63% were living with their parents.

High participation was noted amongst the age group of 18–20 years (58; 23.2%). About 36.4% (91) of respondents had one child; 43.2% (108) had completed secondary school education; 90.4% (226) were Christian; 80.8% (202) were single; 69.6% (174) were unemployed; 62.8 (157) were living with their parents; 12.8% (32) were living with their partners and 85.6% (214) did not have a history of previous abortion (see Table 1).

**TABLE 1:** Demographic characteristics of women seeking termination of pregnancy.

Variables	Number of respondents	Percentage
**Age (years)**		
18–20	58	23.2
21–23	51	20.4
24–26	34	13.6
27–29	37	14.8
30–32	36	14.4
33–35	15	6.0
36–38	11	4.4
39–41	6	2.4
42–44	2	0.8
Total	250	100.0
**Children per respondent**		
0	83	33.2
1	91	36.4
2	47	18.8
3	17	6.8
4	10	4.0
5	2	0.80
Total	250	100.0
**Education level**		
Completed secondary school	108	43.2
Tertiary education	104	41.6
Attending secondary school	23	9.2
No formal education	15	6.0
Total	250	100.0
**Religion**		
Christianity	226	90.4
Other	24	9.6
Total	250	100.0
**Marital status**		
Single	202	80.8
Living with partner	27	10.8
Married	16	6.4
Divorced	4	1.6
Widowed	1	0.4
Total	250	100.0
**Employment status**		
Unemployed	174	69.6
Employed	62	24.8
Self-employed	14	5.6
Total	250	100.0
**Living with**		
Parents	157	62.8
Partners	27	10.8
Children	25	10.0
Alone	20	8.0
Friends or other family member	5	2.0
Husband	16	6.4
Total	250	100.0
**Previous abortions**		
0	214	85.6
1	34	13.6
2	2	0.8
Total	250	100.0

### Respondents’ reasons for requesting termination of pregnancy

A total of 24% of respondents responded with ‘wanting to focus on studies’ as the main reason for requesting TOP at Soshanguve 3 CHC. This was followed by 23% of respondents who reported ‘not being ready to be a parent’. Table 2 presents the participants’ reasons for seeking TOP.

**TABLE 2:** Reasons for seeking legal termination of pregnancy.

Reasons for legal TOP	Number of respondents	Percentage
Wanting to focus on studies	89	24.2
Not being ready to be a parent	85	23.1
Experiencing financial difficulties	80	21.7
Having problems with partner	46	12.5
Not being in a committed relationship	39	10.6
Family is complete	14	3.8
Having medical or health problems	9	2.4
Was pressured into having an abortion	2	0.5
Was sexually assaulted or abused	1	0.3
Travelling to another country for work	1	0.3
Complex with multiple factors	1	0.3
Pregnancy was unplanned	1	0.3

**Total**	**368**	**100**

TOP, termination of pregnancy.

### Comparison of sociodemographic and reasons for termination of pregnancy

A statistical significance has been established between the sociodemographics of women and the reasons for TOP as presented in Table 3.

**TABLE 3:** Comparison of sociodemographics and reasons for termination of pregnancy: Expressed in *p*-values.

Socio-demographic characteristic	Wanting to focus on studies	Not ready to be a parent	Having problems with the partner	Financial difficulties
Age of women	< 0.0001	0.0023	0.0016	0.0002
Women’s level of education	0.0024	-	-	0.0324
Women’s marital status	-	0.0266	-	0.0079
Women’s living arrangement	0.0033	0.0179	-	0.0037

### Respondents’ contraceptive practice

**FIGURE 1:**
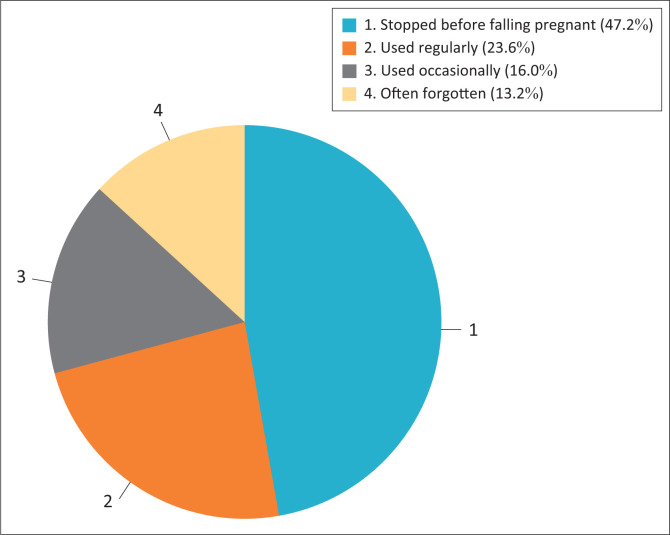
Percentage of contraceptive use by the respondents.

**FIGURE 2:**
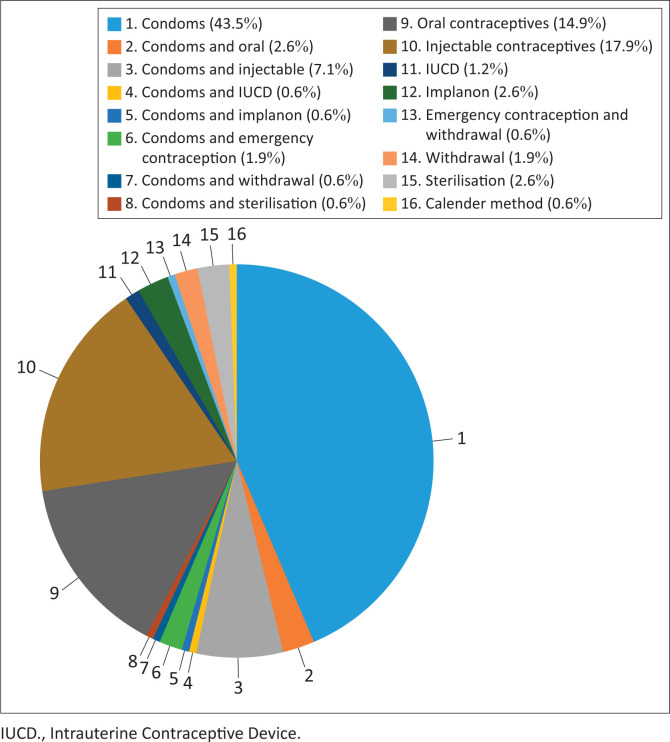
Contraceptive measures used by the respondents.

With regard to contraceptive practice, all 250 respondents confirmed that they had already used contraceptive measures, but some stopped, others used occasionally, others often forgot to use and another group used them regularly as shown in the data in Figure 1. The choice of the method, as revealed by the respondents, varied amongst individuals. Amongst them, 67 (43.5%) had already used a male condom, 27 (17.9%) had used an injectable and 23 (14.9%) had used oral contraceptive (see Figure 2).

### Frequency of contraceptive use as revealed by the respondents

As a result of problems encountered (weight gain, headache, bleeding, dizziness, etc.) while using contraceptives, the majority of women (118; 47.2%) stopped using them, 59 (23.6%) used one regularly, 40 (16%) used one occasionally and 33 (13.2%) often forgot to use.

### Comparison between women’s sociodemographics and methods of contraception

No statistical significance was noted in the comparison between women’s sociodemographics and their methods of contraception as presented in Table 4.

**TABLE 4:** Comparison between women’s sociodemographics and methods of contraception: Expressed in *p*-values.

Sociodemographics	Methods of contraception
Age	0.4219
Level of education	0.4465
Marital status	0.3740
Living arrangement	0.3131

## Discussion

The main findings of this study were that the majority of women at Soshanguve 3 CHC requested TOP for academic reasons. They wanted to focus on and complete their studies before becoming mothers as this would put them in a stronger financial position to care for a child. The majority of these women were younger than 30 years of age, single, had completed secondary school education, were unemployed and living with their parents, and already had one child. They are at an age of building their lives and working on their careers to gain financial independence and stability. This is similar to other studies conducted on women seeking TOP who are aged 20–30 years, unemployed and living with their parents.^4,13,15^ Besides education and not being ready to be a parent, partner-related problems as well as financial difficulties were also reported; this corroborates the causes of lack of preparedness for having a child as established in a previous study by Hammanskraal.^4^ The findings of the current study also match the outcome of a review conducted in 14 countries, where socio-economic concerns were the most mentioned reasons for seeking TOP.^5^

From the study results of 47.2% of women having stopped using contraception, 16% using it occasionally and 23.6% often forgetting to use contraception, it could be inferred that the overall contraceptive practice in the study was unsatisfactory. This is despite the fact that services are free of charge at public clinics and hospitals in South Africa.^16^

In Soshanguve, as well as in Hammanskraal, one common finding amongst all respondents requesting TOP was that they had used contraceptive measures in the past and stopped at some stage along the way.^16^ This attitude had affected their contraceptive practice.

Although the current study did not fully investigate the reasons for unsatisfactory contraceptive practice, other researchers consider that women’s lack of economic empowerment, that is, financial stability, makes it difficult for them to negotiate condom or contraceptive use with a reluctant male partner.^17,18^ This may result in an unwanted, unplanned pregnancy, and a vicious cycle of repeat TOP, which was found to be higher in South Africa than in developed countries.^4^ Some women may go to the extent of keeping their contraceptive use a secret from their male partners who are opposed to contraceptive use in order to control their fertility.^9^

Weight gain, headaches and vaginal bleeding while on hormonal contraceptives were the most common problems experienced by these women. The study did not allow for them to state how they deal with or alleviate these side effects. It is worth noting that most women reported the regular use of contraceptives, particularly condoms, but found themselves with an unplanned and unwanted pregnancy. This calls for more research in the area of condom use to determine the reasons why women fall pregnant while regularly using condoms.

## Limitations

The format of the questionnaire in this study did not allow the researcher to determine issues such as how the respondents manage contraceptive-associated side effects and whether women discontinue contraceptives based on symptoms or tolerate them. This study excluded women less than 18 years of age, who also present for TOP because of high numbers of teenage pregnancy in South Africa. This study had 15 (6%) pregnant women who did not have a formal education (see Table 1).

## Conclusion

While academic reasons, not being ready to be a parent and financial difficulties were named as the main reasons for terminating a pregnancy legally, the selected pregnant women at Soshanguve 3 CHC demonstrated an unsatisfactory practice of contraceptive measures.

## Recommendation

More efforts should be directed towards educating these women on effective use contraceptives.
